# Cycloartane-3,24,25-triol inhibits MRCKα kinase and demonstrates promising anti prostate cancer activity *in vitro*

**DOI:** 10.1186/1475-2867-12-46

**Published:** 2012-11-14

**Authors:** Henry I C Lowe, Charah T Watson, Simone Badal, Ngeh J Toyang, Joseph Bryant

**Affiliations:** 1 Bio-Tech R & D Institute, Kingston, Jamaica; 2 Educational and Scientific Corporation, Florida, USA; 3 Institute of Human Virology, University of Maryland School of Medicine, Baltimore, MD, USA; 4 Natural Products Institute, University of the West Indies, Mona, Jamaica

**Keywords:** Cycloartane-3,24,25 triol, MRCKα kinase, Kinase inhibition, Rostate cancer, Ball moss

## Abstract

**Background:**

Given the high occurrence of prostate cancer worldwide and one of the major sources of the discovery of new lead molecules being medicinal plants, this research undertook to investigate the possible anti-cancer activity of two natural cycloartanes; cycloartane-3,24,25-diol (extracted in our lab from *Tillandsia recurvata*) and cycloartane-3,24,25-triol (purchased). The inhibition of MRCKα kinase has emerged as a potential solution to restoring the tight regulation of normal cellular growth, the loss of which leads to cancer cell formation.

**Methods:**

Kinase inhibition was investigated using competition binding (to the ATP sites) assays which have been previously established and authenticated and cell proliferation was measured using the WST-1 assay.

**Results:**

Cycloartane-3,24,25-triol demonstrated strong selectivity towards the MRCKα kinase with a Kd_50_ of 0.26 μM from a total of 451 kinases investigated. Cycloartane-3,24,25-triol reduced the viability of PC-3 and DU145 cell lines with IC_50_ values of 2.226 ± 0.28 μM and 1.67 ± 0.18 μM respectively.

**Conclusions:**

These results will prove useful in drug discovery as Cycloartane-3,24,25-triol has shown potential for development as an anti-cancer agent against prostate cancer.

## Background

Prostate cancer is the second most frequently diagnosed cancer and sixth leading cause of cancer death in males, worldwide [1]. The search for new molecules to combat the rising cases of prostate cancer especially those resistant to current chemotherapy calls for urgent action. Medicinal plants have been one of the major sources for the discovery of a number of current clinically used anticancer drugs.

**Figure 1:**
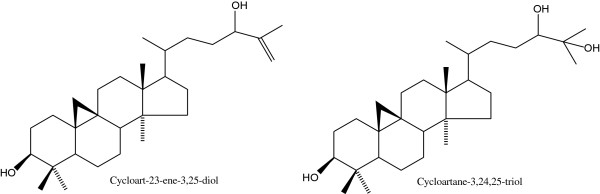
Structures of cycloartanes.

Previous research conducted in our lab demonstrated the promising anti-cancer properties of *Tillandsia recurvata* L. (Bromeliaceae) commonly called the Jamaican Ball Moss or the Old Man’s beard [2]. In an attempt to identify the critical isolate from this plant that is responsible for the observed said bioactivity, this research uncovered the cycloartane, cycloart-23-ene-3,25-diol (Figure 1) [3]. Further literature research led us to a closely related cycloartane, cycloartane-3,24,25-triol (Figure 1), which had previously been extracted from the *Chrysanthemum morifolium* plant and was shown to possess promising chemopreventive properties [4].

Essential biological functions such as the provision of a structural frame work and the driving force for cellular motility and division are mediated by the actin cytoskeleton in all eukaryotic cells. A comprehensive overview of the biological processes that regulate the organization of actin is of recent interest to the regime of cancer therapy [5]. Members of the Rho GTPase family such as myotonic dystrophy kinase-related Cdc42-binding kinases (MRCKα) are key regulators of the actin cytoskeleton and together with multiple target proteins safe guard the tight regulation of normal cell growth and differentiation [6,7]. In the event of genomic alterations or carcinogenesis, eukaryotic cells become predispose to rapid and uncontrollable growth as evidenced by the elevated levels of LIMK1 kinase expressed in prostate cancer, a kinase which itself is activated by MRCKα [7]. It is for this reason that inhibitors of these biochemical entities are thought to restore normal cell proliferation and provide a key solution to cancer treatment. The aim of this study therefore was to confirm the promising in vitro anti prostate cancer activity of 2 cycloartane isolates and their effect on prostate cancer related kinases, namely MRCKα.

## Results and discussion

GTPases are activated by GTP exchange factors (GEFs) and down-regulated by GTPase-activating proteins (GAPs). Prolonged activation of these molecular entities can initiate a series of signal transduction pathways that can impinge on a number of cellular processes [7]. Amongst the transducers of these signals are protein kinases and increase Rho signalling has been associated with tumour development and/or progression from various origins [8]. The MRCKα kinase, an effector of CDC42 GTPase with functional overlap with ROCK1/2, has also been suggested to have a role in tumour progression [9] and/or invasion [10].

Given the reported elevated levels of MRCKα in cancer tumours relative to normal neighbouring tissues [9,10], the potent selective inhibitory (Kd_50_ = 0.26 μM) properties of cycloartane-3,24,25-triol towards the MRCKα kinase of a total of 451 kinases tested makes it a promising candidate for further investigation into the treatment of prostate cancer. Not only did this isolate inhibit the MRCK kinase but it also reduced the viability of the PC-3 cell line with an IC_50_ value of 2.226 ± 0.28 μM (Table 1). This indicates that cycloartane-3,24,25-triol is more effective than other promising leads towards this cell line as Terracciano et al., [11] investigated an inhibitor of this cell line and only 38% inhibition was observed at 4 μM. In addition to reducing the viability of the PC-3 cell line, cycloartane-3,24,25-triol also reduced the viability of another prostate cancer cell line DU145 with an IC_50_ value of 1.67 ± 0.18 μM (Table 1). This observed bioactivity makes this isolate even more relevant in prostate cancer treatment than omega-3FAs; docosaheenois acid (DHA) and eicosapentaenoic acid (EPA) that both reduced the viability of PC-3 cell line comparable to cycloartane-3,24,25-triol but showed no impact towards DU145 cells [12].

**Table 1:** **IC**_**50**_**results of WST-assay for PC-3 and DU145**

**Antiproliferation activity (IC**_**50**_**: μM)**		**Kinase inhibition (Kd**_**50**_**μM)**
**PC-3**	**Du145**	**MRCKα**
2.226 ± 0.28	1.67 ± 0.18	**0.26**

## Conclusions

We report for the first time this inhibition of MRCKα kinase inhibition by cycloartane-3,24,25-triol. Of the 451 kinases, cycloartane-3,24,25-triol selectively inhibited MRCKα with greater potency than other potential chemotherapeutics. Our findings show that the bioactivity of this isolate not only inhibits MRCKα known to be associated with prostate cancer, but also reduced the viability of two prostate cancer cell lines, PC-3 and DU145 implicating the validity of these results in anti-cancer drug discovery.

## Material and methods

### Chemicals

The Cayman’s WTS-1assay kit which was used for the cell proliferation assay and kinase inhibition assay chemicals were purchased from Cayman Chemical Company.

### Cell lines and culture medium

All cell lines with their respective media and supplements were obtained from ATCC (Manassas, VA, USA).

### Cell proliferation

The cells (PC-3 and DU145) were maintained in minimum essential media (MEM) supplemented with 10% foetal calf serum (FCS), 20 mM l-glutamine, 2% penicillin–streptomycin, and 0.2% gentamicin. Cells were maintained at 37°C with 5% CO_2_ in Corning 75 cm^3^ culture flasks. Cells were trypsinized and plated at the appropriate density (500–2000 cells/well in log phase 72 h post drug addition) into 96 well plates in media for approximately 18 h after which they were exposed to cycloartanes; cycloart-23-ene-3,25-diol and cycloartane, cycloart-23-ene-3,25-triol for 72 h. The compounds were solubilized in DMSO (>0.1%). Following the appropriate treatments, cell proliferation was measured using the WST-1 (4-[3-(4-iodophenyl)-2-(4-nitrophenyl)-2H-5-tetrazolio]-1, 3-benzene disulfonate) (Roche) colorimetric assay according to the manufacturer’s instructions [13]. All assays were performed in duplicates and were monitored spectrophotometrically at 450 nm/690 nm (Synergy HT 96-well Plate Reader - BIO-TEK). Cell viability was assessed as percent of drugs relative to vehicle solvent-treated control. IC_50_ values were determined from the extract dose versus control growth curves using Graph Prism software.

### Kinase inhibition assay

**Figure 2:**
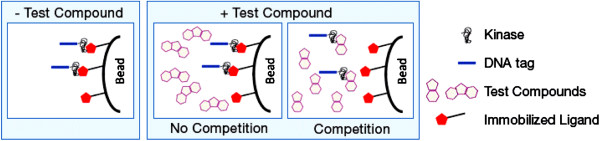
How the KinomeScan assay works (Courtesy of DiscoveRx).

Competition binding assays were established, authenticated and executed as described previously [14,15]. For most assays, kinases were fused to T7 phage strains [14] and for the other assays, kinases were produced in HEK-293 cells after which they were tagged with DNA for quantitative PCR detection (data not shown). In general, full-length constructs were used for small, single domain kinases, and catalytic domain constructs for large multi-domain kinases. The binding assays utilized streptavidin-coated magnetic beads treated with biotinylated small molecule ligands for 30 minutes at room temperature which generated affinity resins for the kinase assays. The liganded beads were blocked with excess biotin and washed with blocking buffer (SeaBlock (Pierce), 1% BSA, 0.05% Tween 20, 1 mM DTT) to remove unbound ligand and to reduce non-specific phage binding. Binding reactions were assembled by combining kinases, liganded affinity beads, and test compounds in 1x binding buffer (20% SeaBlock, 0.17x PBS, 0.05% Tween 20, 6 mM DTT). Test compounds were prepared as 40x stocks in 100% DMSO and diluted directly into the assay (Final DMSO concentration = 2.5%). All reactions were performed in polypropylene 384- well plates in a final volume of 0.04 ml. The assay plates were incubated at room temperature with shaking for 1 hour and the affinity beads were washed with wash buffer (1x PBS, 0.05% Tween 20). The beads were then re-suspended in elution buffer (1× PBS, 0.05% Tween 20, 0.5 μM non-biotinylated affinity ligand) and incubated at room temperature with shaking for 30 minutes. The kinase concentration in the eluates was measured by quantitative PCR and Table 1 presents kd_50_ for kinase interaction with the test compound. These were determined using a standard dose response curve using the hill equation. Curves were fitted using a non-linear least square fit with the Levenberg-Marquardt algorithm. Figure 2 illustrates the kinase interaction process.

### Plant material: *Tillandsia recurvata* (extraction and isolation)

The whole *T. recurvata* plant was collected from trees and electricity poles at Kingston, Jamaica. A voucher specimen of the plant was identified at the Institute of Jamaica Herbarium where it is deposited with Accession Number: IJ 3411. The collected plant material was air dried, pulverized into powder. 2.3 kg of Ball Moss biomass was extracted twice with 5 L of Chloroform. The filtrate was dried in a rotavapor to obtain a dark green residue (87.6 g).

## Competing interest

The authors declare no competing interest at this time.

## Authors’ contribution

Conceived and designed the experiments: HL and JB, Acquisition of data: CW and NT, Analyzed and interpreted the data: NT and SB. Wrote the paper: SB and NT, Critical and intellectual revision of the article contents: SB and NT. All authors read and approved the final manuscript.

## Authors’ information

Dr. Henry Lowe is a specialist in medicinal chemistry with approximately 50 years of experience in the field, he currently holds a PhD from the Manchester University, a M,Sc. from the University of Sydney and B.Sc. (hons) from UCWI, London University. He holds several postdoctoral studies including but not limited from; Harvard University and M.IT, USA. Dr. Simone Badal is currently a post-doctoral fellow at the Natural Products Institute, UWI, Mona where she recently completed her PhD (Biochemistry) in cancer research and holds a B.Sc. (hons) and M.Phil. in Biochemistry from the University of the West Indies, Mona. She also obtained her MBA in International Relations from the University of Wales, Cardiff. Ms. Charah Watson is a PhD candidate specialising in Natural products chemistry in the Department of Chemistry at the UWI, Mona. Her research activities include natural product isolation, elucidation and identification and their use as natural pest control and anti-cancer agents. Mr. Ngeh Toyang is a PhD candidate in Pharmacognosy with the Division of Pharmacognosy, Leiden University where his research focus is on cancer and HIV/AIDS. Dr. Joseph Bryant holds a Doctor of Veterinary Medicine degree from Tuskegee University specialising in cancer and HIV/AIDS research. A board certified Laboratory Animal Veterinarian with a background in Comparative Medicine, Dr. Bryant joined IHV from the National Institutes of Health.
